# Non-Melanoma Skin Cancers and Other Cutaneous Manifestations in Bone Marrow Failure Syndromes and Rare DNA Repair Disorders

**DOI:** 10.3389/fonc.2022.837059

**Published:** 2022-03-10

**Authors:** Jennie Vagher, Amanda Gammon, Wendy Kohlmann, Joanne Jeter

**Affiliations:** Family Cancer Assessment Clinic, Huntsman Cancer Institute, University of Utah, Salt Lake City, UT, United States

**Keywords:** non-melanoma skin cancer, cancer predisposition syndromes, bone marrow failure syndromes, skin cancer genetics, genomic instability disorders

## Abstract

Although most non-melanoma skin cancers are felt to be sporadic in origin, these tumors do play a role in several cancer predisposition syndromes. The manifestations of skin cancers in these hereditary populations can include diagnosis at extremely early ages and/or multiple primary cancers, as well as tumors at less common sites. Awareness of baseline skin cancer risks for these individuals is important, particularly in the setting of treatments that may compromise the immune system and further increase risk of cutaneous malignancies. Additionally, diagnosis of these disorders and management of non-cutaneous manifestations of these diseases have profound implications for both the patient and their family. This review highlights the current literature on the diagnosis, features, and non-melanoma skin cancer risks associated with lesser-known cancer predisposition syndromes, including bone marrow failure disorders, genomic instability disorders, and base excision repair disorders.

## Introduction

Non-melanoma skin cancers are very common, but their hereditary risk factors receive little attention. In the general population, incidence of basal cell and squamous cell cancers varies significantly depending on geographic location, which is tied to levels of ultraviolet (UV) radiation exposure and population demographics. A systematic review of non-melanoma skin cancer incidence reported basal cell carcinoma incidence of 76.21/100,000 person-years and squamous cell carcinoma incidence of 22.65/100,000 person-years in England (1). By contrast, the reported incidences from Australia were markedly higher (884/100,000 for basal cell carcinoma & 387/100,000 person-years for squamous cell carcinoma). Data from both countries also showed marked regional differences (1) However, due to the high incidence rate and the low potential for metastatic disease, comprehensive data on non-melanoma skin cancer is often not recorded in cancer statistic databases such as the Surveillance, Epidemiology, and End Results Program (SEER) or GLOBOCAN (2, 3). This practice makes estimating its true worldwide incidence difficult (1, 2). While UV radiation exposure remains the primary risk factor for cutaneous non-melanoma skin cancer, this review highlights multiple genetic conditions that elevate the risk of skin cancer.

Of note, our review does not cover Nevoid Basal Cell Carcinoma syndrome (NBCCS) (also known as Gorlin syndrome or basal cell nevus syndrome (BCNS)). NBCCS is associated with a high lifetime risk for basal cell carcinoma (~90% of individuals with NBCCS) as well as an elevated risk for medulloblastoma. Multiple comprehensive reviews have been published on NBCCS, including Bresler 2016, as well as updates to management guidelines (4, 5). This review will synthesize the current literature on non-melanoma skin cancer risk in the following cancer predisposition syndromes: Telomere Biology Disorders, Fanconi anemia, Xeroderma pigmentosum, Bloom syndrome, Werner syndrome, Rothmund-Thompson syndrome, and Ferguson-Smith syndrome. This review will provide an overview of syndromic manifestations, incidence of skin cancers, and treatment/surveillance implications with the purpose of increasing provider awareness and correct diagnosis.

## Bone Marrow Failure Syndromes

Telomere biology disorders and Fanconi Anemia are the most common bone marrow failure syndromes. Their causes lie in telomere maintenance and DNA repair, significantly disrupting the body’s ability to protect itself from cancer development. Affected individuals have characteristic dermatologic features and experience elevated risks for young onset non-melanoma skin cancers, in addition to other risks for solid tumors and hematologic malignancy.

## Telomere Biology Disorders

The telomere biology disorders (TBDs) constitute a spectrum of clinical disorders arising from short telomeres. Professor Ferdinand Zinsser is widely credited with the first publication on dyskeratosis congenita (DC), a subtype of the TBDs, in 1910 (6, 7). Classic DC is associated with the triad of oral leukoplakia, nail dystrophy, and reticulated skin pigmentation. However, since discovery of the *DKC1* gene in 1999, 13 more genes have been identified that cause short telomeres, leading the designation of TBDs as a reflection of the fundamental biology that unites these disorders (8, 9). In general, TBDs predispose individuals to develop bone marrow failure, acute myeloid leukemia/myelodysplastic syndrome (AML/MDS), interstitial lung disease, liver cirrhosis, and/or features of premature aging (i.e. early greying of the hair). Cutaneous manifestations associated with TBDs include both phenotypic manifestations, such as hyperkeratosis and/or reticulated skin pigmentation, as well as an increased risk to develop cutaneous carcinomas. Due to the changing landscape of TBDs, underdiagnosis of TBDs remains a challenge and the overall incidence is unknown. It is estimated that the classic subtype of TBDs, DC, has an incidence of 1 in 1,000,000, though DC accounts for less than 5% of all TBDs (10). The most common cause of mortality is bone marrow failure, followed by pulmonary complications and cancer (10).

### Mechanism

Telomeres consist of double stranded TTAGGG nucleotide repeats at the ends of eukaryotic chromosomes, which protect these chromosomal ends from normally occurring DNA damage signaling and repair activities created by DNA breaks (11). Telomere length varies considerably within different populations, and telomere shortening is a normal consequence of aging (11). With every cell division, telomere lengths decrease. Eventually a critical threshold is reached, triggering cellular senescence, and some cells may then undergo apoptosis (10, 11).

The telomerase complex, including telomere reverse transcriptase (TERT) and its RNA component (TERC), is responsible for adding nucleotide repeats to telomeric ends (12). The shelterin subunits and complex, including protection of telomeres 1 (POT1), are involved in creating and stabilizing the T-loop structure, preventing DNA damage response, recruiting telomerase, and regulating telomere elongation (10). The CST (CTC1-STN1-TEN1) complex and other proteins play a role in telomere maintenance. Other reviews have been published on the mechanisms of telomeres (11).

### Genes/Inheritance

Pathogenic germline variants (PGV) in 14 genes have been described as the cause for the TBDs, including *TERT*. Variants in these genes can be inherited in x-linked recessive, autosomal dominant, and autosomal recessive patterns; however, it is also possible for an individual to have a TBD due to a *de novo* PGV. There is considerable genetic heterogeneity, and most of the genes associated with TBDs have associations with multiple TBD subtypes and inheritance patterns. For example, DKC1 is associated with x-linked recessive disease, but PGV in this gene can be seen in individuals with classic DC features as well as those who present in infancy with Hoyeraal-Hreidarsson syndrome (HH). PGV in genes such as *RTEL1*, *TINF2*, and *ACD* are associated with autosomal dominant (heterozygous) risks for pulmonary fibrosis or bone marrow failure as well as autosomal recessive (homozygous or compound heterozygous) risks for HH. Variants in *NAF1, TERT, TERC, PARN*, and *RTEL1* have been associated with clinical presentations of pulmonary fibrosis, liver disease, or bone marrow failure in middle or later age (8). In individuals who have autosomal dominant disease, genetic anticipation affecting disease severity in successive generations has been reported (13, 14).

### Means of Diagnosis

The diagnosis of TBDs is complex. Though genetic testing can aid in making the diagnosis of a TBD, it is not the gold standard. Individuals needing diagnostic testing are recommended to undergo flow cytometry with fluorescent *in situ* hybridization (flow FISH) in leukocyte subsets (15). Flow FISH is the only clinical validated test proven for TBD diagnosis (16). Total lymphocyte telomeres measured to be less than the 1^st^ centile for age are 97% sensitive and 91% specific with a positive predictive value of 85% for the diagnosis of a TBD (15). Accelerated telomere shortening is induced by exposure to intensive chemotherapies, which can affect the results of telomere length measurement testing (17). More studies are needed to determine the effect of different treatments on telomere attrition.

Genetic testing, once very short telomeres are identified, complements clinical evaluations and telomere length measurement testing. Genetic testing can also be useful to help determine inheritance patterns, to identify genotype-phenotype correlations, and to offer to family members seeking knowledge of disease status. Approximately 20-30% of individuals diagnosed with classic DC do not have an identifiable PGV, which means a substantial number of patients who have a diagnosable TBD *via* telomere length measurement testing do not have a PGV and a diagnosis may be missed if relying on genetic testing alone (18).

### Population/Epidemiology

The overall incidence of TBDs cannot be accurately estimated. DC has an incidence of 1 in 1,000,000, though DC accounts for less than 5% of all TBDs (10). It is estimated that 41% of familial mixed hematologic and interstitial lung disease and 3% of familial hematologic disorders are due to TBDs (19). As with most genetic predispositions, it is likely that TBDs are underdiagnosed, which contributes to the difficulty in calculating incidence.

### Clinical Characteristics: Cutaneous

TBDs are associated with the classic mucocutaneous triad of nail hypoplasia, reticulated skin pigmentation, and oral leukoplakia. Though this triad is diagnostic, it may be absent or progressive in individuals, leading to underdiagnosis. In a publication of 60 individuals with DC, 37% had the complete clinical triad, while 10% lacked all triad features (8, 20). Other cutaneous findings have been reported such as adermatoglyphia (loss of fingerprints), palmoplantar hyperkeratosis, hyperhidrosis, premature graying, scalp/eyelash hair loss, epiphora, and lash irritation (20, 21). Individuals who are carriers of *DKC1* PGVs may have some features of the mucocutaneous triad, but largely remain asymptomatic.

### Clinical Characteristics: Non-Cutaneous

Bone marrow failure (aplastic anemia) may be the first presenting feature of TBDs, and the risk is highest for individuals who have classic DC (about 80-90% lifetime risk) (22). Risks to develop AML and MDS are most significant over age 50. In a recent publication, approximately 18 of 180 patients with a TBD were diagnosed with AML or MDS (23). Other hematologic conditions have been described, such as macrocytosis, isolated cytopenias, paroxysmal nocturnal hemoglobinuria and essential thrombocythemia (24).

### Incidence of Skin Cancer

Cutaneous squamous cell carcinomas (cSCCs) have been reported in 1.5% of individuals with TBDs and are diagnosed at young ages. There is a significantly increased risk to develop mucosal SCCs, especially in the buccal mucosa, nasopharynx, esophagus, rectum, vagina, and cervix (25). For the purpose of this review, we will focus on cSCCs; other publications have reported on risks for mucosal SCCs in the TBD population (10, 18). A summary of publications reporting on non-melanoma skin cancer in chronological order will be included below.

Data from the United Kingdom Dyskeratosis Congenita Registry (DCR) in 2000 reported 148 patients with TBDs and eight solid tumors amongst the patients. There was a single patient reported with cutaneous carcinoma unspecified at age 20 years. It is unclear if the patient with cutaneous carcinoma in this series was male or female and whether this was a pre-transplant diagnosis (22).

A literature review and quantitative analysis from the National Cancer Institute’s Inherited Bone Marrow Failure Syndrome (NCI IBMFS) was published in 2009. The literature review (1910-2008) reported 60 solid tumors in 51 patients. This included eight patients with cSCCs, diagnosed at a median age of 21 with a range of diagnosis between age 4 and 43. A male predominance was reported (7 males and 1 female). All of these skin carcinomas were reported prior to hematopoietic stem cell transplant (HSCT). The quantitative analysis (2002-2008) included 50 patients. Of these patients, there was a single case of cutaneous carcinoma and this patient was diagnosed with BCC one year after HSCT at age 29 (26, 27).

Another literature review in 2010 included 560 cases of TBDs in the literature. Of these patients, six total cSCCs were reported, with five being reported in male patients. The median age of diagnosis was 18, and the range was age four to 34 (28).

Data from NCI IBMFS in 2018 included 197 TBD patients from 108 families. This analysis (2002-2016) reported 14 SCC and six BCC in nine patients. Of these nine patients, 5 of these patients reported two or more skin cancers (2 patients with two each, 2 with three each, and one with five). An additional skin cancer (age and subtype unknown) was reported in a TBD patient during the development of the manuscript, bringing the total skin cancers in this cohort to 10. The age of skin cancer diagnoses ranged from 14 to 54 with a median age of 35 at diagnosis (18).

Other registry TBD cohorts exist, though some registries have intentionally excluded cutaneous cancer diagnoses and it is unclear if cutaneous cancers were intentionally excluded in others due to limitations in methodology (23, 29).

### Surveillance and Management Considerations: Cutaneous

Thorough skin examinations with a dermatologist are recommended at least annually, though starting age for screening is not specified (21, 30). Often SCC arises from leukoplakic plaques, and dermatology exams are recommended to focus on mucosal and genital areas. Surveillance for head and neck SCC is recommended to start at age 16 (31). According to data presented, age of cSCCs ranged from age four to age 54 with median ages of 18, 21, and 35 from the NCI IBMFS and literature review data sets (18, 26–28). The data is limited regarding the number of cSCCs reported in those under age 18, so there are gaps in our understanding of whether skin exams would be beneficial in adolescence. In other cancer predisposition syndromes, the youngest known age of a diagnosis provides a guide of when to start surveillance, so for TBDs, age four would be the youngest diagnosis and thus an appropriate age to start skin exams (28).

Prevention strategies are also recommended, including limiting sun exposure, using sunscreen regularly, using facial moisturizer with sunscreen every day, wearing hats and sun protective clothing, avoiding tanning beds, awareness of reflected sun off of snow and water, and performing regular self-skin exams (10, 21). To the authors’ knowledge, no discussion of best treatment for non-melanoma skin cancers has been proposed.

### Surveillance and Management Considerations: Non-Cutaneous

Published guidelines for the management of TBDs suggest baseline surveillance and annual surveillance depending on clinical presentation. In addition to dermatologic screening as discussed above, individuals are recommended to undergo evaluation for risks relating to hematology, pulmonology, immunology, otolaryngology, and gastroenterology, among other specialty providers. Bone marrow failure is typically treated with bone marrow transplant. Recommendations can be complex given the needs of this patient population, so referral to a center that has expertise in the management of bone marrow failure syndromes is recommended (21).

## Fanconi Anemia

Fanconi anemia (FA) is a heterogeneous syndrome associated with DNA repair defects. FA is associated with risks for bone marrow failure, acute myeloid leukemia/myelodysplastic syndrome, congenital abnormalities, endocrine anomalies, and/or solid cancers. Dr. Guido Fanconi’s characterization of three brothers in a family in 1927 lead to the naming of FA, though it was not for another 40 years that the mechanism of spontaneous chromosomal breakage was identified as the etiology of FA (32, 33). The cutaneous manifestations associated with FA include both phenotypic manifestations, such as café au lait macules, as well as an increased risk to develop cutaneous carcinomas. The most common solid tumor in individuals with FA are head and neck squamous cell carcinomas (HNSCC), but Wilms tumor and medulloblastoma are also seen. FA is primarily an autosomal recessive condition. FA affects 1 in 100,000 births in the USA, and there are well known founder effects causing higher incidences amongst populations outside of the USA. The main causes of death are cancer, bleeding, infection, and complications from hematopoietic stem cell transplant (HSCT) (34). Survival has improved in high resource countries due to a reduction in mortality by bleeding or infection complications, leading to a need for increased awareness of skin cancer risks in this population (35).

### Mechanism

The FA pathway assists in DNA repair, DNA replication, and other cellular processes. A principal function of this pathway is to remove a critical barrier, interstrand crosslink lesions (ICL), which interfere with DNA replication and genetic transcription and must be repaired or bypassed for cells to survive (36). Both nucleotide excision repair and homologous recombination pathways are required to make major repairs to ICLs (37). Each human cell has to repair approximately 10 interstrand crosslinks per day, and between 20-40 lesions can lead to cell death (38, 39). Interstrand crosslinks are generated both exogenously and endogenously, and when unrepaired, they lead to DNA breakage and chromosomal rearrangements, which lead to the development of cancer (39, 40). The FA pathway is the only known mechanism of repair for interstrand crosslink lesions (41). There is also increasing evidence that subsets of FA proteins contribute to other pathways (37, 41).

Specifically relating to non-melanoma skin cancers, a recent publication has highlighted alternative mechanisms to explain an increased risk for skin cancers suggesting that individuals with FA have inherent subclinical dermatologic vulnerabilities causing the significant risk to develop SCC (42). The authors hypothesize that inherited structural defects of the FA epidermis (and potentially mucosa) cause vulnerabilities to mechanical and/or environmental stress, thus further compounding the existing genome instability to promote SCC (42). The FA pathway is essential in the developing epidermis for sustained keratinocyte adhesion and appropriate restraint of proliferation (42).

### Means of Diagnosis

Cells from patients with Fanconi Anemia are extremely sensitive to ICL-generating agents, such as mitomycin C (MMC) or diepoxybutane (DEB). Thus, the gold standard test for the diagnosis of FA is a chromosome breakage analysis (CBA) performed on a blood sample. Genetic testing can aid in the diagnosis of FA, but is typically only performed after a CBA. CBA may not be definitive due to mosaicism in peripheral blood, hypomorphic variants, and other chromosomal instability disorders that mimic FA (43). Interpretation of a CBA should made with a genetics provider familiar with BMF syndromes and testing, especially if there is a high level of concern for underlying FA.

### Genes/Inheritance

There are over 20 genes that have been implicated in the etiology of FA (42). All pathogenic variants in these genes are associated with autosomal recessive disease with the exception of *FANCB* (x-linked) and *FANCR* (autosomal dominant). The majority of FA is associated with pathogenic variants in the *FANCA* gene (44). Importantly, most individuals who are carriers for FA do not have any associated risks with being carriers; rather, determining carrier status is most important for family planning. A portion of individuals who are carriers for FA will have an increased risk to develop adult-onset cancers, such as breast or ovarian cancers. Pathogenic variants in *BRCA2/FANCD1, PALB2/FANCN, BRIP1/FANCJ*, and *RAD51C/FANCO* have known adult-onset cancer risks, and there are recommendations for carriers to have increased cancer surveillance, consider chemoprevention, or undergo preventative surgeries (45).

### Population/Epidemiology

FA affects 1 in 100,000 births in the USA, with a carrier frequency of approximately 1 in 181 (range 1:156-1:209) in the USA (46). There are known founder effects in genetically isolated populations, causing a birth incidence in Afrikaners of 1 in 22,000 (1:88) and in Israel of 1 in 45,000 (1:93) (46, 47). The Spanish Gypsy population has the highest carrier frequency for FA with a founder *FANCA* variant (1:64-1:70) while a significant portion (1%) of Bantu speaking Black individuals in Southern Africa have a founder *FANCG* variant (48, 49).

### Clinical Characteristics: Cutaneous

Pigmentary changes of the skin have been reported by case series as the most common (up to 68%) cutaneous skin feature in FA. These pigmentary changes include cafe-au-lait macules (CALMs), flexural hyperpigmentation, and hypopigmented macules (32, 50). In a recent review of cutaneous features in FA, at least one cutaneous feature was present in 97% of participants with the most common being CALMs. Both a combination of diffuse hyperpigmented and hypopigmented macules and a combination of CALMs with hypopigmented macules are cutaneous features that may help to differentiate FA from other genetic syndromes with similar presentations (50).

### Clinical Characteristics: Non-Cutaneous

Cytopenia and bone marrow failure are the most common presenting features in FA. Most individuals with FA will develop a cytopenia progressing to bone marrow failure. Thrombocytopenia with red blood cell macrocytosis and elevated fetal hemoglobin often lead to the diagnosis of FA (51). Individuals with FA present with bone marrow failure at various times in life, and the literature suggests 75% present within the first decade of life (28, 52, 53). Adults with FA do not typically present with bone marrow failure, but rather due to a diagnosis of cancer or severe toxicity after chemotherapy for treatment of a malignancy (52–54). There are risks to develop AML and MDS, both in childhood and adulthood (55, 56).

### Incidence of Skin Cancer

Cutaneous squamous cell carcinomas (cSCCs) have been reported in individuals with FA, though the exact risk to develop cSCCs in FA is unknown. There is a significantly increased risk to develop mucosal SCCs, especially in the buccal mucosa, nasopharynx, esophagus, rectum, vagina, and cervix (25). For the purpose of this review, we will focus on cSCCs (and other non-melanoma skin cancers), as other publications have reported on risks for mucosal SCCs in the FA population (57, 58). A summary of publications reporting on non-melanoma skin cancers in chronological order will be included below.

The International Fanconi Anemia Registry (IFAR) in 2003 reported on 754 individuals with FA. This included three individuals who reported cSCCs. Median and age range of diagnoses were not available (53).

Data from the National Cancer Institute’s Inherited Bone Marrow Failure Syndrome (NCI IBMFS) in 2010 included 66 patients with FA. In this analysis (2002-2008), two diagnoses of cSCCs at age 33 and 38 and one diagnosis of BCC at age 31 were reported (27).

In 2016, the Italian Fanconi Anemia Registry reported 180 individuals with FA. Of these patients, 20 developed solid tumors. This included one individual with a diagnosis of unspecified skin cancer. Age of diagnosis was not available. This patient was reported to have multiple tumors, including a genital tract, breast, and head/neck tumor (34).

Data from the NCI IBMFS in 2018 included 163 individuals with FA. In this analysis (2002-2016), 23 SCC and 12 BCC were diagnosed amongst 11 patients. The median age of diagnosis was 33 years old, and the range of diagnosis was from age 26 to 41. Eight patients reported at least two skin cancers (six patients with two, one with three, and one with 17 separate skin cancers). An additional unspecified skin cancer (age unknown) was reported in an FA patient during the development of the manuscript, bringing the total skin cancers in this cohort to 36 (18).

A recent study from a German Bone Marrow Failure Registry in 2021 reported 421 individuals with FA. Of these patients, 33 patients developed cancer. This included one individual with a skin carcinoma NOS at age 15 prior to the development of AML at age 16. Notably in this cohort, cancer risks into adulthood (over age 17) were not ascertained, which limits cancers reported and the true estimate of cutaneous carcinomas may be underrepresented due to the age cutoff (59).

Data from a Cincinnati-based FA cohort in 2021 included 105 individuals with FA. Of these patients, nine patients reported a history of cutaneous SCC or BCC. There was no median age of diagnosis nor any range of diagnoses reported, though all of these patients were adults when they were included in the publication (between ages 24 to 51). There was one case of melanoma in this publication. Age of diagnosis was not included, but the site of the lesion was on the breast. Three of the patients included were post-transplant, including a patient who reported 50+ cSCCs (42).

Other FA cohort publications exist, but do not include any cases of non-melanoma skin cancers (28, 29, 60). An interesting note is that melanoma is rarely reported in individuals with FA. A hypothesis has been proposed that the FA pathway may be protective against melanoma tumorigenesis in patients with FA (61).

### Surveillance and Management Considerations: Cutaneous

Total body skin examinations are recommended annually starting at the age of 18. Despite the recommendation to start surveillance in young adulthood, preventative behaviors for sun protection and avoidance should be implemented at an early age. These include limiting sun exposure, using sunscreen regularly, wearing hats and sun protective clothing, avoiding tanning beds, and performing regular self-skin exams (51).

In the general population, therapies such as topical chemotherapy agents (5-Fluorouracil) and other drugs that stimulate an immune response to target precancerous lesions or cancers may be utilized to treat non-melanoma or melanoma skin cancers. The tolerability and efficacy of these agents in FA have not been well-studied. In this population, the best curative option to treat non-melanoma skin cancers and melanoma remains surgical removal (51).

### Surveillance and Management Considerations: Non-Cutaneous

Guidelines for the management of FA are published through the Fanconi Anemia Research Foundation (34). Guidelines suggest baseline surveillance and annual surveillance depending on clinical presentation. In addition to the dermatologic screening detailed above, individuals are recommended to undergo evaluation for risks relating to hematology, immunology, otolaryngology, and gastroenterology, among other specialty providers. Cancer or aplastic anemia treatment may require dose reduction depending on chemotherapy utilized as well as avoidance of use of radiation therapy. Bone marrow failure often requires hematopoietic stem cell transplant. Recommendations can be complex given the needs of this patient population, so referral to a center that has expertise in the management of bone marrow failure syndromes is recommended.

## Genomic Instability Syndromes

The following three syndromes, Bloom, Werner, and Rothmund-Thomson, share multiple features. Their causes lie primarily in the genes which encode for the RecQ family of helicases. Lack of these helicase proteins results in genomic instability, causing disruption in multiple body processes, including aging, growth, immunity, cell resistance to UV radiation, and DNA repair. Affected individuals have characteristic dermatologic features and experience elevated risks for young onset non-melanoma skin cancers, sometimes with unusual histologies or locations.

## Bloom Syndrome

In 1954, dermatologist Dr. David Bloom first reported on three individuals with similar features suggesting a syndrome: growth deficiency, unique erythematous lesions on the face over the cheeks and nose (butterfly rash), and significant sun sensitivity leading to additional skin lesions on the lips, eyes, and other sun exposed areas (62). This syndrome now bears Dr. Bloom’s name and is also associated with immunodeficiency, increased cancer risks, lung disease, diabetes, and fertility concerns (premature menopause/male infertility). Much of the available research on Bloom syndrome comes from the Bloom Syndrome Registry, which was started in 1960 at Weill Cornell Medicine (Cornell University) (http://www.med.cornell.edubsr/).

### Mechanism

Bloom syndrome results from a lack of functional BLM protein. BLM is a DNA helicase responsible for restricting sister chromatid exchanges (SCE). BLM is in a family of RecQ helicases that includes WRN and RECQL4, responsible for Werner syndrome and Rothmund-Thompson syndrome, respectively. Absence of functional BLM leads to significantly increased rates of SCE during DNA replication, which leads to elevated genomic instability. Injecting a line of Bloom syndrome cells with cDNA containing working copies of BLM results in normal rates of SCE (63). BLM’s helicase activity is also directly involved with the double stranded DNA break repair process through interaction with DNA2, RPA, and the Mre11-Rad50-Nbs1 (MRN) complex (64).

### Genes/Inheritance

Bloom syndrome is an autosomal recessive condition caused by biallelic pathogenic variants in the *BLM* gene, also called *RECQL3*. Most *BLM* pathogenic variants result in protein truncation (63). In an evaluation of the 64 different *BLM* pathogenic mutations reported by individuals in the Bloom Syndrome registry, 54 were variants leading to protein truncation and 10 were missense variants (65). Backers et al. recently described an individual with clinical Bloom syndrome who had a deep intronic variant in *BLM* (c.3020-258A>G, intron 15) along with a nonsense variant in exon 18 (called c.3379C>T, p.Gln1127Ter) on the opposite allele (66).

### Means of Diagnosis

The very high level of SCE seen in Bloom syndrome cells is pathognomonic for the condition. SCE analysis was used for many years to confirm a diagnosis of Bloom syndrome (67). Today molecular genetic testing of *BLM* to assess for biallelic mutations is the initial test when Bloom syndrome is suspected clinically.

### Population/Epidemiology

Bloom syndrome is very rare, with less than 300 affected individuals reported in the Bloom syndrome registry. A higher incidence of Bloom syndrome is seen in the Ashkenazi Jewish population due to the presence of a common founder variant (called c.2207_2212delinsTAGATTC). Studies estimate between 1 in 37 to 1 in 100 individuals of Ashkenazi Jewish decent are heterozygous carriers of this pathogenic variant in *BLM* (68, 69). A less common founder variant in the Ashkenazi Jewish population is c.2407dupT.

### Clinical Characteristics: Cutaneous

The red “butterfly” rash (erythema and telangiectasias) seen on the cheeks, nose, eyelids, and ears of individuals with Bloom syndrome typically develops within the first year of life (67, 70, 71). This rash is correlated with the onset of sun exposure for the affected child. The rash can also affect the arms and hands and can worsen with continued sun exposure (67, 71). Individuals with Bloom syndrome also have elevated rates of café-au-lait macule development and areas of hypopigmentation (67, 70, 71). Cheilitis and poikiloderma can also be present (67, 71).

### Clinical Characteristics: Non-Cutaneous

Patients with Bloom syndrome typically show growth deficiency prenatally and into adulthood (70). Attainment of developmental milestones and cognitive factors are not typically impacted by Bloom syndrome (70). Individuals can experience immunodeficiency. While infections can be frequent, they are not often severe (72). Impaired fertility can be seen in both women and men with Bloom syndrome. Most men reported with Bloom syndrome are infertile and many women experience early menopause (67, 71, 73). Risks for endocrine dysfunction, particularly diabetes and hypothyroidism, are also elevated (67).

A range of non-cutaneous malignancies have been reported in Bloom syndrome. Leukemia and lymphoma are the most common, followed by colon and breast cancers (67). Using data from the 277 individuals enrolled in the Bloom syndrome registry at that time, Cuniff et al. reported that 33.4% developed cancer by age 25 and 80.9% developed cancer by age 40 (67).

### Incidence of Skin Cancer

Within the Bloom syndrome registry data, basal cell carcinoma is the most frequently reported cutaneous cancer (13 reported basal cell carcinomas among 277 registry patients) (67). Mean age at basal cell carcinoma diagnosis was 28 (range 18-38) (67). Only 4 squamous cell carcinomas were reported (age range 35-36), as well as 4 undefined skin cancers (67). No melanoma diagnoses are known among patients enrolled in the Bloom syndrome registry (67).

### Surveillance and Management Considerations: Cutaneous

Individuals with Bloom syndrome are recommended to limit sun exposure and utilize sun protective behaviors (including frequent application of SPF 30 sunscreen and use of protective clothing). Annual dermatology exams are also recommended, although no specific starting age is specified (67).

### Surveillance and Management Considerations: Non-Cutaneous

Comprehensive management recommendations for individuals with Bloom syndrome based on data from the Bloom Syndrome Registry can be found in Cuniff et al. (67). In terms of cancer surveillance, individuals with Bloom syndrome are recommended to undergo annual colonoscopy, with FIT stool testing every 6 months, starting at age 10-12 (31, 67). While a moderately increased risk for colon cancer has been described in heterozygous carriers of a single pathogenic *BLM* variant, data has remained mixed on this association and no changes to colorectal cancer screening are currently recommended (74, 75). Annual breast MRI is recommended to begin at age 18 for individuals at elevated breast cancer risk (31, 67). Due to concerns about ionizing radiation conferring an increased risk for secondary malignancy, screening imaging should utilize MRI or ultrasound whenever possible (67). Individuals with Bloom syndrome should be alert for signs of leukemia and lymphoma, including increasing pallor, fatigue, enlarging lymph nodes, abnormal bleeding or weight loss, and petechiae (67). Screening for lymphoma *via* whole body MRI every 1-2 years is recommended to begin at age 12-13 (67). For Wilms tumor, abdominal ultrasound every 3 months until age 8 is recommended, along with awareness of symptoms (abdominal mass, blood in the urine) (67).

Treatment of malignancies in patients with Bloom syndrome is complicated by increased sensitivity to radiotherapy and chemotherapy, leading to elevated adverse reactions and secondary malignancies (67, 76). Reduction in chemotherapy dosage (suggested 50% or lower) has been recommended (67). Use of radiation therapy should be avoided whenever possible (67).

Immunodeficiency screening is recommended for anyone with Bloom syndrome who experiences recurrent infection or is being treated with immunosuppressive drugs (67). Growth hormone treatment can also be considered to potentially impact growth deficiency (67).

## Werner Syndrome

Dr. Otto Werner first detailed this syndrome in 1904 (77, 78). The primary clinical presentation of Werner syndrome is of premature aging. The first sign of Werner syndrome can be slowed growth starting around puberty, although many affected individuals are not diagnosed until their late 30s-40s (79). Additional symptoms of Werner syndrome, including bilateral cataracts, thinning of the skin, and hair graying, generally present in a person’s 20s. Median life expectancy for individuals with Werner syndrome is age 54, with cancer and atherosclerosis driving mortality (77, 79, 80). The International Werner Syndrome Registry at the University of Washington (https://dlmp.uw.edu/research-center/werner/registry) and the Japanese Werner Consortium at Chiba University are the leading sources of research on Werner syndrome.

### Mechanism

Werner syndrome results from the myriad effects of increased genomic instability due to lack of function of the WRN protein. WRN has both helicase and exonuclease functions (79, 81). It is involved with multiple factors in the base excision repair and double strand break DNA repair pathways (82). WRN is also believed to assist with telomere maintenance and to interact with intricate DNA formations during transcription and replication (79, 83, 84). Laarmann et al. showed that functional WRN is necessary for full endothelial cell motility; they propose that lack of WRN function may lead to the increased atherosclerosis risks in Werner syndrome in multiple ways, due to detrimental impacts on blood vessel repair and inflammation (82).

### Genes/Inheritance

Werner syndrome is an autosomal recessive condition caused by biallelic pathogenic variants in *WRN* (81). As of 2017, a total of 83 different pathogenic variants in *WRN* have been described, per data from the International Werner Syndrome Registry and Japanese Werner Consortium, plus published literature (79). A complete list of these variants is available at http://www.pathology.washington.edu/research/werner/database/. The most frequent WRN pathogenic variant in the Japanese population is c.3139-1G>C, which has been reported to account for 70.7% of WRN pathogenic variants in the Japanese Werner Consortium (79). Two additional WRN variants are relatively common in Japan, c.1105C>T and c.3446delA (79). Most pathogenic variants in *WRN* lead to protein truncation, although a few missense variants have also been reported that disrupt protein function (79).

### Means of Diagnosis

Werner syndrome can be clinically diagnosed using a set of criteria proposed by the International Registry of Werner Syndrome (84). The cardinal signs are defined as: 1) bilateral cataracts, 2) premature graying/thinning of the scalp hair, 3) specific dermatological features (tight and atrophic skin, skin ulceration, hyperkeratosis, subcutaneous atrophy (regional), altered pigmentation, and “bird-like” facies), and 4) short stature. Additional signs of Werner syndrome include: diabetes, flat feet, osteoporosis, osteosclerosis of distal phalanges of fingers/toes, soft issue calcification, vocal changes, premature atherosclerosis or heart attack, and hypogonadism (84). To have a definite diagnosis of Werner syndrome, individuals need to have all four cardinal signs, plus two additional signs. A probable diagnosis of Werner syndrome occurs in patients with bilateral ocular cataracts, premature graying/thinning of the scalp hair, and the dermatological features outlined above plus two additional signs (84). Identification of biallelic pathogenic *WRN* variants can help further distinguish a diagnosis of Werner syndrome from other syndromes which can cause features of premature aging.

### Population/Epidemiology

While individuals with Werner syndrome have been identified worldwide, the majority of reported cases of Werner syndrome (approximately 60%-80%) have been identified in Japan (Takeomoto & Yokote 2020). In Japan, approximately 3 in 100,000 individuals have Werner syndrome (2005 Shibuya).

### Clinical Characteristics: Cutaneous

Development of skin ulcers is a primary source of morbidity for individuals with Werner syndrome. Approximately 40% of individuals with Werner syndrome develop skin ulceration (85). They most commonly develop at pressure points on the leg, ankle, soles of the feet, and elbows (86).

Skin thinning, tightness, and dryness are common signs of premature dermatological aging in Werner syndrome (85). Patients can be prone to developing calluses, which if not treated, can progress to skin ulcers (85). Poikiloderma has also been reported in patients with Werner syndrome (85).

### Clinical Characteristics: Non-Cutaneous

Individuals with Werner syndrome develop multiple features of aging significantly earlier than the general population, including early hair graying, type II diabetes, cataracts, and arteriosclerosis (77). Of note, an increased risk for dementia or age-related cognitive impairment has not been reported in Werner syndrome (84). It is estimated that 55% of individuals with Werner syndrome develop type II diabetes, with a mean age of onset in the late 30s (87). Osteoporosis has been documented in approximately 41% of patients with Werner syndrome (88).

The most common non-cutaneous cancers associated with Werner syndrome include thyroid cancer (particularly follicular type), soft-tissue sarcomas, leukemia/pre-leukemia/lymphoma, and osteosarcoma (77, 80). The estimated median age for cancer diagnosis for patients with Werner syndrome living in Japan is 44.5 years (80). An elevated incidence of meningioma development (both benign and malignant) has also been reported in Werner syndrome (80). A systemic review of cancer incidence reported in Werner syndrome found that 22% of individuals developed multiple primary malignancies (80).

### Incidence of Skin Cancer

Non-melanoma skin cancer (basal and squamous cell) has been reported in individuals with Werner syndrome. In the systemic review noted above, non-melanoma skin cancer accounted for 4.8% of all reported malignancies in patients with Werner syndrome (12 cases among 189 patients) (80). An earlier review of malignancies reported in Werner syndrome between 1939 – August 1995 identified 10 cutaneous non-melanoma cancers out of a total 186 reported neoplasms (89).

Individuals with Werner syndrome have an elevated risk for melanoma, particularly uncommon forms of melanoma. Shibuya et al., reported on a 44 year-old woman with Werner syndrome who developed three primary malignant melanomas (one labial, two on sole of the left foot) (77). They identified 26 other reported individuals in the literature with Werner syndrome and melanoma, including two other patients with multiple primary melanomas (77). Of note, 15 of the reported malignant melanomas were acral while 10 occurred intranasal (77). Lauper found a standardized incidence ratio (SIR) for melanoma in patients with Werner syndrome of 53.5 compared to the general Osakan population (80).

### Surveillance and Management Considerations: Cutaneous

Treatment and management of skin ulcers is a key concern for patients with Werner syndrome. Topical medications and dressings are primarily recommended for treatment of skin ulcers (86). Surgical intervention with skin grafts can be considered for ulcers that are resistant to healing (86). Annual dermatology exam is recommended for patients starting at the time of diagnosis with Werner syndrome to assess for calluses, ulcers, and malignancies (90). Special care should be taken during the dermatology exam to carefully examine any area suspicious for an ulcer or callus as cutaneous malignancies have been reported in the same areas as well (86). Concerns regarding adverse effects from chemotherapy for malignancies (cutaneous and non-cutaneous), including elevated immunosuppression and potential for secondary malignancies, have been raised for patients with Werner syndrome, although few examples of adverse effects of treatment have been reported to date (77, 80).

### Surveillance and Management Considerations: Non-Cutaneous

A comprehensive update to management recommendations for Werner syndrome was recently published in the journal Geriatrics and Gerontology International (91). Bringing together Werner syndrome experts, the authors created consensus recommendations for the treatment and management of primary Werner syndrome features, including dyslipidemia and fatty liver, sarcopenia, diabetes, osteoporosis, infection, skin ulcers, and tendon calcifications.

## Rothmund Thomson Syndrome

Rothmund-Thomson syndrome (RTS) was first described in 1868 by ophthalmologist Auguste Rothmund (92). It is characterized by dermatological features (rash progressing to poikiloderma), bilateral juvenile cataracts, short stature, dystrophic hair/nails, elevated risk for cancer (particularly osteosarcoma), and increased risk for infertility. Cognition is typically unaffected. Malignancy is a significant cause of mortality in RTS, but lifespan can be otherwise normal (93). Rothmund-Thomson has been classified into two types, with Type II being the most common. Patients with Type I RTS do not appear to have the same elevated cancer risks or skeletal anomalies seen in many individuals with Type II, but do develop bilateral cataracts (which is not generally seen with RTS II) (94, 95).

### Mechanism

Rothmund-Thomson syndrome results from loss of functional RECQL4 or ANAPC1. RECQL4 is a DNA helicase in the same family as WRN and BLM (96). REQL4 appears to be involved in double stranded DNA repair through stabilization of the Mre11-Rad50-Nbs1 complex; absence of functional RECQL led to premature degradation of the MRN complex *in vitro* (97). APC1 is a ubiquitin ligase and the protein coded for by *ANAPC1.* APC1 is key to appropriate movement of cells through the cell cycle, in particular the transition from metaphase to anaphase, as well as regulation of DNA replication (94, 98).

### Genes/Inheritance

Rothmund-Thomson syndrome (RTS) is an autosomal recessive condition most often caused by pathogenic variants in *RECQL4*. Recently, pathogenic variants in *ANAPC1* have been associated with autosomal recessive RTS Type I (94). Pathogenic variants in *RECQL4* are associated with RTS Type II.

### Means of Diagnosis

The pattern of poikiloderma development in early childhood is highly characteristic of Rothmund-Thomson syndrome and distinct from the butterfly rash that develops in Bloom syndrome. Genetic testing of REQL4 and ANAPC1 can also assist in confirming a diagnosis of RTS. Approximately 30% of individuals affected with RTS will not have identifiable pathogenic variants in *REQL4* or *ANAPC1* (93).

### Population/Epidemiology

Rothmund-Thomson is a very rare hereditary condition, with fewer than 500 cases reported in the literature (93, 95). Carrier frequency is currently unclear.

### Clinical Characteristics: Cutaneous

The typical initial presenting symptom of Rothmund-Thomson syndrome is a facial rash (most often presenting between ages 3-6 months, although can start later up to age 2) (99). This rash can spread to the buttocks and later develops into poikiloderma. Poikiloderma describes a collection of cutaneous features include skin atrophy, telangiectasias, and areas of hyper/hypo pigmentation.

### Clinical Characteristics: Non-Cutaneous

Osteosarcoma risk is particularly elevated in young individuals with RTS, with the median age of diagnosis being 10 (31). Among a cohort of 41 patients with RTS described by Wang et al., 32% developed an osteosarcoma (99). Skeletal anomalies including altered or missing thumbs, ulnar defects, or patellar hypoplasia (93). Osteopenia requiring endocrine management can occur, as well as infertility (99).

### Incidence of Skin Cancer

Both basal cell and squamous cell carcinoma have been reported in individuals with RTS. Due to the rarity of RTS, lifetime risk for non-melanoma skin cancers in the RTS population is unclear, but believed to be elevated over the general population with a trend toward younger age at onset (100–102). Stinco et al. reported a history of multiple cutaneous malignancies in a man with RTS and reviewed published reports of cutaneous malignancies in RTS (102). In total, they identified reports of 61 patients with RTS in the literature who developed at least one malignancy. Among these 61 patients, 11 developed squamous cell carcinoma (including 3 patients with SCC of the tongue), 3 developed basal cell carcinoma (including one woman who developed 11 basal cell carcinomas between the ages of 46-63), two had Bowen’s disease, and one had a verrucous carcinoma (102).

The following reports were included in the review by Stinco et al. Piquero-Casals et al. reported on three siblings with RTS Type II with cutaneous squamous cell carcinoma (100). One sibling was diagnosed with a verrucous carcinoma of the foot at age 38 and a squamous cell carcinoma (SCC) of the hand at age 48 (100). Another sibling was diagnosed with squamous cell carcinoma at the base of the thumb at age 41. The third affected sibling was diagnosed with an SCC after biopsy of an ulcer on his left heel at age 35. All three SCCs were HPV negative (100).

### Surveillance and Management Considerations: Cutaneous

Annual dermatology exams are recommended for individuals with RTS to screen for skin cancers and provide treatment for skin concerns (no starting age for the exams is specified) (31). Treatment of telangiectasias for cosmetic concerns can be completed using pulsed dye laser treatment. Reported treatment of skin cancers in affected patients was largely through excision (with skin grafts when necessary) (102).

### Surveillance and Management Considerations: Non-Cutaneous

A baseline skeletal survey is recommended for patients with RTS before age 5 to look for skeletal anomalies (31). Annual ophthalmology exams are also recommended to screen for cataract development, although an age to start is not specified (31).

## Xeroderma Pigmentosum

Xeroderma Pigmentosum (XP) was first described in 1874 by Moriz Kaposi in the dermatology textbook he wrote with Ferdinand von Hebra. XP is characterized by extreme photosensitivity. Average lifespan is reduced in individuals with XP, with the median age at death being between 29-37, depending on the presence or absence of neurological involvement (103). Skin cancer is the most common cause of mortality, followed by neurological impairment and non-cutaneous malignancies (104).

### Mechanism

XP is most often caused by problems in the nucleotide excision repair pathway, which is responsible for repairing damage to skin cells from UV exposure (105). Eight different complementation groups were initially defined for XP and are now linked to individual genes (with the exception of *ERCC1*) (103).

### Genes/Inheritance

XP is an autosomal recessive condition. To date, biallelic pathogenic variants in nine genes have been associated with XP: *DDB2*, *ERCC1*, *ERCC2*, *ERCC3*, *ERCC4*, *ERCC5*, *POLH*, *XPA*, and *XPC*.

### Means of Diagnosis

XP is typically clinically diagnosed based on dermatologic features, with genetic testing used to confirm a diagnosis and provide additional phenotype/genotype information.

### Population/Epidemiology

A number of founder pathogenic variants have been identified worldwide, including IVS3-1G>C in *XPA* in Japan (carrier frequency of 1 in 100) (106, 107). In part due to the existence of multiple founder pathogenic variants across numerous countries and differences in consanguinity rates among populations, prevalence of XP differs among countries. Likewise, the proportion of XP attributable to different genes varies from country to country (**Table 1** summarizes data from the US) (104). In the US and Europe, approximately 1 in 1,000,000 individuals have XP. In Japan, approximately 1 in 22,000 people are affected with XP (107). Increased prevalence has also been reported in North Africa and the Middle East.

**Table 1:** Characteristics of Cancer Predisposition Syndromes Associated with Non-Melanoma Skin Cancers.

Disorder (Incidence of disorder)	Mode of diagnosis	Genes associated	Inheritance pattern	Percent of syndrome associated with PV in gene
Fanconi Anemia (108)	Chromosome breakage analysis *via* Mitomycin C chromosome breakage study (MMC) and diepoxybutane (DEB) assay	*FANCA*	AR	60%
		*FANCB*	XL	2%
		*FANCC*	AR	12%
		*BRCA2 (FANCD1)*	AR	2%
		*FANCD2*	AR	2%
		*FANCE*	AR	2%
		*FANCF*	AR	2%
		*FANCG*	AR	10%
		*FANCI*	AR	<2%
		*BRIP1 (FANCJ)*	AR	<2%
		*FANCL*	AR	Rare
		*FANCM*	AR	Rare
		*PALB2 (FANCN)*	AR	Rare
		*RAD51C (FANCO)*	AR	Rare
		*SLX4 (FANCP)*	AR	Rare
		*ERCC4 (FANCQ)*	AR	Rare
		*RAD51 (FANCR)*	AD	Rare
		*BRCA1 (FANCS)*	AR	Rare
		*UBE2T (FANCT)*	AR	Rare
		*XRCC2 (FANCU)*	AR	Rare
		*REV7 (FANCV)*	AR	Rare
Telomere biology disorders (109)	Telomere length measurement *via* flow FISH	*DKC1*	XLR	20-25%
		*TINF2*	AD, AR	12-20%
		*TERC*	AD, AR	5-10%
		*TERT*	AD, AR	1-7%
		*NOP10*	AD, AR	<1%
		*NHP2*	AD, AR	<1%
		*CTC1*	AR	1-3%
		*RTEL1*	AD, AR	2-8%
		Unknown		20-30%
Bloom syndrome (110)	Clinical features (“butterfly rash”), genetic testing	*BLM*	AR	100%
Werner Syndrome (90)	Clinical diagnostic criteria, genetic testing	*WRN*	AR	Near 100%
Rothmund-Thomson (93)	Clinical features (poikiloderma pattern), genetic testing	*ANAPC1*	AR	10%
		*RECQL4*	AR	60%
Ferguson-Smith syndrome	Clinical features of numerous MSSE, genetic testing	*TGFBR1*	AD	Not available
Xeroderma Pigmentosum (104)	Severe photosensitivity, genetic testing	*DDB2*	AR	3% (US)
		*ERCC1*	AR	Rare (US)
		*ERCC2*	AR	28% (US)
		*ERCC3*	AR	1% (US)
		*ERCC4*	AR	0% (US)
		*ERCC5*	AR	3% (US)
		*POLH*	AR	7% (US)
		*XPA*	AR	9% (US)
		*XPC*	AR	43% (US)

AD, autosomal dominant; AR, autosomal recessive; MSSE, multiple self-healing squamous epithelioma; PV, pathogenic variant; XL, X-linked; XLR, X-linked recessive.

### Clinical Characteristics: Cutaneous

XP causes extreme UV light sensitivity. Approximately 60% of affected individuals will develop blistering sunburns with UV light exposure, while 40% have the ability to tan rather than burn (104). There is evidence for a genotype/phenotype correlation with the tendency to burn/blister in the sun. Patients considered to have XP complementation group C (associated with pathogenic variants in XPC), report higher levels of tanning rather than burning on sun exposure, while patients with XP complementation groups A or D (associated with *XPA* or *ERCC2* respectively) reported more frequently burning easily in the sun (104). It has been noted that XP patients who tend to tan rather than burn are often diagnosed with younger onset skin cancers, which may be due to less frequent sun protective behaviors (104). Nearly all individuals with XP develop small pigmented, freckle-like macules on the skin in areas of UV exposure (face, back of neck, back of hands, upper chest) in early childhood (106). Skin erythema and development of actinic keratoses are common in areas of UV exposure.

### Clinical Characteristics: Non-Cutaneous

It is estimated that 25%-30% of individuals with XP have neurological symptoms, including worsening cognitive impairment, absent or reduced deep tendon reflexes, sensorineural hearing loss, and acquired microcephaly (106). In patients with neurological complications from XP, hearing loss can start in childhood and complete loss may be apparent around age 15 (106). Difficulties with walking can also start in the pre-teen years and progress to needing ambulatory assistance (such as wheelchair use) by approximately age 15 (106). Joint contractures and difficulties with feeding and respiration can present in mid-late teenage years as well. Neurodegeneration can be seen on CT and MRI with evidence of brain atrophy in multiple areas (106).

### Incidence of Skin Cancer

Individuals with XP have high risks for young onset non-melanoma and melanoma skin cancers. For patients under the age of 20, XP results in an approximate 10,000-fold increase in non-melanoma skin cancer risk and a 2,000-fold increase in melanoma risk (104). In reviewing the records of all 106 patients with XP admitted to the NIH between 1971-2009, 64 (60%) had been diagnosed with non-melanoma skin cancer (104). Age at non-melanoma skin cancer diagnosis ranged from 1-32 years, with a median age of 9. Of the 38 patients with a melanoma diagnosis, 33 (87%) of them also had a history of non-melanoma skin cancer (104) Age at first melanoma diagnosis ranged from 2-47 years, with the median age at 22 years (104). Many individuals had a history of numerous skin cancer diagnoses (104). One specific patient had a history of 284 basal cell carcinomas, 12 squamous cell carcinomas, and 24 melanomas (all verified with pathology records) (104). Mucocutaneous oral cancers are also seen more frequently in individuals with XP, due to UV exposure in this area. Squamous cell carcinoma of the tongue tip was reported by Wade and Plotnick in two patients with XP (111). Saleh and Elansary reported the presence of an oral angiokeratoma on the tongue of a 20 year-old male patient with XP (112).

### Surveillance and Management Considerations: Cutaneous

The most imperative management recommendation for individuals with XP is to avoid UV exposure (31). Use of sunscreen and protective clothing (including long sleeves, long pants, hats, scarves, gloves, visors, and sunglasses) while outside during the day is imperative (31, 106). UV exposure within the home should be reduced through using low UV emission lightbulbs (such as LED) and applying UV protection to windows (31). Dermatology exams are recommended every 3 months, beginning at age of diagnosis (31).

Treatment of skin cancers is ideally completed with surgical excision (106). Radiation treatment of malignancies should be avoided (31).

### Surveillance and Management Considerations: Non-Cutaneous

Ophthalmology exams are recommended every 3-6 months to monitor for ocular complications (106). Frequent auditory and neurological evaluations are recommended (31, 106). The needs of patients with neurological symptoms for supportive interventions/aides should be monitored. Hearing and mobility aides, as well as later intensive assistance with feeding and respiration, can be necessary (106)

## Ferguson-Smith Syndrome

Among the syndromes discussed in this review, Ferguson-Smith syndrome is unique. It appears to only predispose to cutaneous findings, which typically resolve on their own, but can be significantly disfiguring. J Ferguson-Smith reported the first documented case in a 23 year-old male in 1934 at a meeting of the North British Dermatological Society (113). This condition is very rare, with just over 100 cases reported worldwide. The primary cutaneous manifestation of Ferguson-Smith syndrome is the development of multiple self-healing squamous epitheliomas (MSSE).

### Mechanism

Ferguson-Smith syndrome results from the loss of TGFBR1. The product of *TGFBR1* acts as a tumor suppressor and shows loss of heterozygosity in MSSE cells (114, 115).

### Genes/Inheritance

Ferguson-Smith is an autosomal dominant condition resulting from pathogenic variants in *TGFBR1.* Pathogenic variants in *TGFBR1* are also associated with a separate condition, Loeys-Dietz syndrome. Loeys-Dietz syndrome is a connective tissue disorder causing an increased risk for aortic aneurysm. Affected individuals characteristically have bifid uvula, hypertelorism, and arterial tortuosity (116). Loss of function pathogenic variants causing a dominant negative effect are proposed to cause Loeys-Dietz syndrome while Ferguson-Smith syndrome appears to result from pathogenic variants causing haploinsufficiency (116, 117). Reported pathogenic variants in *TGFBR1* resulting in Ferguson-Smith syndrome are truncating or missense variants (114).

### Means of Diagnosis

No formal clinical diagnostic criteria for Ferguson-Smith syndrome have been published. Diagnosis is made based on dermatological examination and identification of multiple MSSE (biopsy proven). Genetic testing of *TGFBR1* can assist in further confirming a diagnosis.

### Population/Epidemiology

The first reported case of Ferguson-Smith syndrome and most subsequent families have been identified in Scotland (113, 118).

### Clinical Characteristics: Cutaneous

The primary cutaneous manifestation of Ferguson-Smith syndrome is the development of multiple self-healing squamous epitheliomas (MSSE). These lesions typically present on the face and extremities (arms, legs) (113). MSSE appear very similar to invasive squamous cell carcinomas, yet heal spontaneously over time (119). Development of MSSEs typically starts in an affected individual’s late teens/20s.

### Clinical Characteristics: Non-Cutaneous

Individuals with Ferguson-Smith syndrome are not known to have an increased risk for other health concerns unrelated to MSSE.

### Surveillance and Management Considerations: Cutaneous

Surgical removal of MSSE from regions of the skin at elevated risk for squamous cell cancer development is recommended. However, due to the usually benign nature of MSSE, extensive surgery is to be avoided if possible.

## Conclusion

The diagnosis of non-melanoma skin cancers in individuals at early ages and/or with other syndromic features presents an opportunity to identify cancer predisposition syndromes. Though non-melanoma skin cancers may not be the most prominent feature of many of the syndromes reviewed in this article, there is a need for dermatology and oncology providers to be aware of these conditions nonetheless. The cutaneous malignancies and other cutaneous features associated with these syndromes is summarized in **Table 2**, and additional online resources for these syndromes are given in **Table 3**. Cutaneous malignancies for people with these conditions tend to be treated surgically, as for cutaneous non-melanoma skin cancers in the general population. However, appropriate testing and accurate diagnosis for these cancer predisposition syndromes are critical for management of associated health risks for these patients and their family members.

**Table 2:** Malignant and Non-Malignant Cutaneous Findings in Cancer Predisposition Syndromes.

Syndrome	Non-malignant cutaneous features	Non-melanoma skin cancer associations	Melanoma associations
Telomere Biology Disorders	Reticulated skin pigmentation, dermatoglyphia, palmoplantar hyperkeratosis, hyperhidrosis, premature graying, scalp/eyelash hair loss, epiphora, and lash irritation	Squamous cell carcinoma (SCC) and basal cell carcinoma (BCC)	None reported
Fanconi Anemia	Café au lait macules, hypopigmented macules and patches, skinfold freckle-like macules	SCC and BCC	Only one reported patient with melanoma
Bloom Syndrome	Erythematous rash over nose, cheeks, eyelids, lips “butterfly rash”	BCC (most common), some SCC reported	None reported
Werner Syndrome	Prematurely aging skin (thinning, tight)	SCC and BCC	Significantly elevated risk
Rothmund-Thomson Syndrome	Poikilodermas, sensitivity to UV radiation	SCC and BCC	
Xeroderma Pigmentosum	Extreme UV radiation sensitivity, blistering sunburns	Significantly elevated risk for BCC and SCC	Significantly elevated risk
Ferguson-Smith Syndrome	Multiple self-healing squamous epitheliomas (MSSE)	MSSE can appear similar to SCC	None reported

UV, ultraviolet.

**Table 3:** Resources for Selected Cancer Predisposition Syndromes.

Disorder	Support group or resource name	Support group	Research/Registry	Educational resource	Financial support available	URL
Fanconi Anemia	Fanconi Anemia Research Fund	X	X	X	X	https://www.fanconi.org/
	Genereviews			X		https://www.ncbi.nlm.nih.gov/books/NBK1401/
Telomere Biology Disorders	Team Telomere	X	X	X	X	https://teamtelomere.org/
	Genereviews			X		https://www.ncbi.nlm.nih.gov/books/NBK22301/
Bloom Syndrome	Bloom Syndrome Registry (Weil Cornell)		X	X		http://www.med.cornell.edu/bsr/
	Bloom Syndrome Association (NH)	X	X	X	X	https://www.bloomsyndromeassociation.org/
Werner Syndrome	International Werner Syndrome Registry (Univ of WA)		X	X		https://dlmp.uw.edu/research-center/werner/registry
	Japanese Werner Syndrome Consortium		X			Not currently available online
Rothmund-Thomson	RTS Foundation		X	X		https://www.rtsplace.org
Xeroderma Pigmentosum	Xeroderma Pigmentosum Society (NY)	X		X		https://www.xps.org/
	XP Support Group (UK)	X		X	X	https://xpsupportgroup.org.uk/

## Author Contributions

JV, AG, WK, and JJ contributed to conception and design of the manuscript. AG and JV wrote the first draft of the manuscript. All authors contributed to manuscript revision, read, and approved the submitted version.

## Funding

This publication utilized the Genetic Counseling Shared Resource at Huntsman Cancer Institute at the University of Utah and was supported by the National Cancer Institute of the National Institutes of Health under Award Number P30CA042014.

## Author Disclaimer

The content is solely the responsibility of the authors and does not necessarily represent the official views of the NIH.

## Conflict of Interest

The authors declare that the research was conducted in the absence of any commercial or financial relationships that could be construed as a potential conflict of interest.

## Publisher’s Note

All claims expressed in this article are solely those of the authors and do not necessarily represent those of their affiliated organizations, or those of the publisher, the editors and the reviewers. Any product that may be evaluated in this article, or claim that may be made by its manufacturer, is not guaranteed or endorsed by the publisher.
